# Three new pyrrole alkaloids from the endophytic fungus *Albifimbria viridis*

**DOI:** 10.1007/s13659-022-00327-2

**Published:** 2022-02-24

**Authors:** Pan-Pan Wei, Jia-Cheng Ji, Xu-Jun Ma, Zheng-Hui Li, Hong-Lian Ai, Xin-Xiang Lei, Ji-Kai Liu

**Affiliations:** School of Pharmaceutical Sciences, South-Central University for Nationalities, Wuhan, 430074 People’s Republic of China

**Keywords:** Pyrrole alkaloids, *Coptis chinensis*, Endophytic fungus, *Albifimbria viridis*, Immunosuppressive activity

## Abstract

Three new pyrrole alkaloids albifipyrrols A–C (**1–3**), were isolated from the endophytic fungus *Albifimbria viridis* collected from the Chinese medicinal plant. Their structures were elucidated by extensive NMR and HRESIMS spectrometric analyses. All compounds were evaluated for immunosuppressive activity. Fortunately, compound **2** exhibits certain inhibition specifically against the LPS-induced proliferation of B lymphocyte cells with IC_50_ value 16.16 μM.

**Graphical Abstract:**

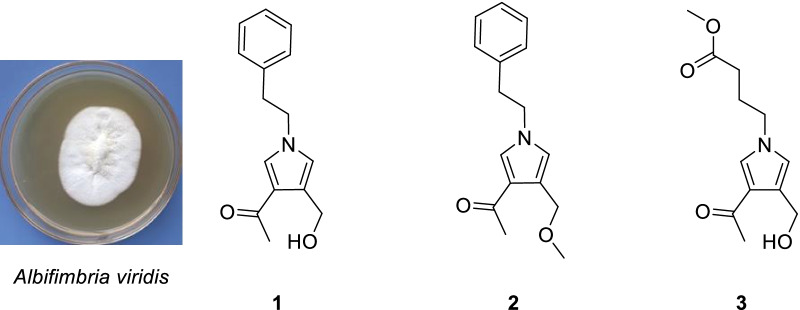

**Supplementary Information:**

The online version contains supplementary material available at 10.1007/s13659-022-00327-2.

## Introduction

The human immune system is a complex network of defensing against foreign invaders. Autoimmune diseases arise when the immune system fails to distinguish between self and non-self [1, 2]. Immunosuppressants are often used to prevent and treat the immune rejection of organs and tissues of transplant patients and play an important role in the treatment of various autoimmune diseases [3–8]. Nevertheless, some common immunomodulatory drugs such as mycophenolate mofetil (MMF) and cyclosporin A (CsA) have low efficacy, toxicity, and serious side effects in transplant patients [9–12]. Therefore, it is necessary to find more efficient, safe and novel immunosuppressants to improve rejection.

In recent years, endophytic fungi from plants have been widely regarded as a significant source of drugs [13]. A great quantity of compounds with novel structures and multiple bioactivities are constantly isolated [14–16]. For instance, the well-known anticancer drug paclitaxel can be produced from Pacific yew by the endophytic fungus *Taxomyces andreance* [17]. *Coptis chinensis* Franch is a famous Chinese medicine in China. Modern pharmacological and clinical studies have indicated that it has anti-tumor, anti-inflammatory, antibacterial, hypoglycemic and other pharmacological activities [18–20]. However, there are few reports on endophytic fungus of *C. chinensis* Franch.

**Fig. 1:**
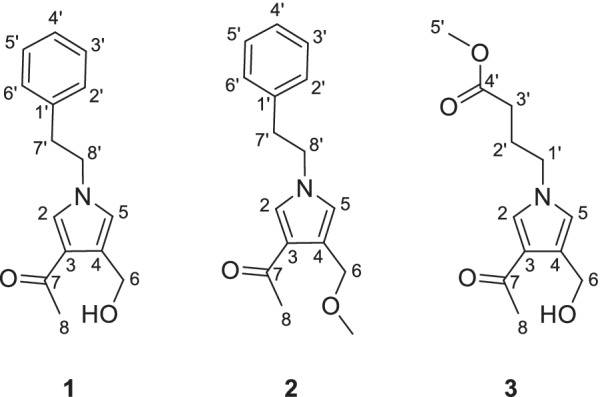
The chemical structures of compounds **1–3**

During the past few years, we had the aim of finding new potential immunosuppressive agents from endophytic fungus of *C.chinensis* Franch. Fortunately, we obtained a pyrrole alkaloid with immunosuppressive activity from *Albifimbria viridis*. Herein, we report the details of the isolation, structure elucidation, and bioactivities of three pyrrole alkaloids albifipyrrols **A–C** (**1–3**) (Fig. 1).

## Results and discussion

**Fig. 2:**
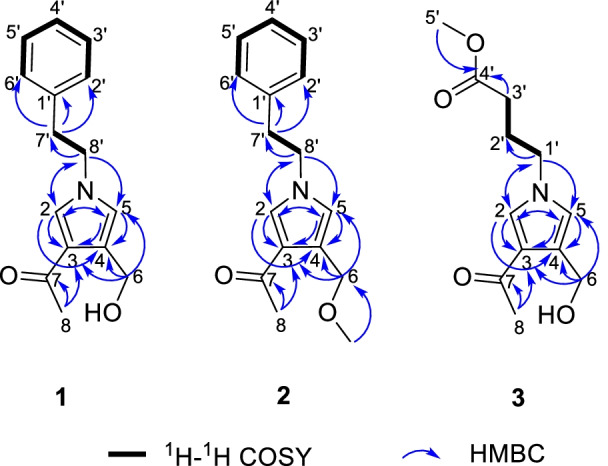
Key HMBC and ^1^H-^1^H COSY correlations of compounds **1**–**3**

Compound **1** was obtained as yellow oil. The molecular ion peak of HR-ESI–MS was at *m/z* 266.1150 [M + Na]^+^ (calcd for 266.1157), which indicated that the molecular formula of compound **1** is C_15_H_17_NO_2_, with eight degrees of unsaturation. In the ^1^H-NMR spectrum (Table 1), a monosubstituted benzene moiety at *δ*_H_ 7.13 (2H, d, *J* = 7.8 Hz, H-2′, H-6′), 7.24–7.28 (2H, m, H-3′, H-5′) and 7.18–7.22 (1H, m, H-4′), two mutually coupling aromatic protons at *δ*_H_ 6.72 (1H, d, *J* = 2.2 Hz, H-5) and 7.40 (1H, d, *J* = 2.2 Hz, H-2), two heteroatom-bearing methylenes at *δ*_H_ 4.56 (2H, s, H-6) and 4.16 (2H, t, *J* = 7.1 Hz, H-8′), one conventional methylene at *δ*_H_ 3.06 (2H, t, *J* = 7.1 Hz, H-7′) and one methyl group at *δ*_H_ 2.32 (3H, s, H-8) were clearly shown. The ^13^C-NMR and DEPT spectrum (Table 1) of **1** showed the presence of fifteen carbons, including one methyl, three methylenes [including two heteroatom-bearing methylenes at *δ*_C_ 58.5 (C-6), 52.5 (C-8′)], seven aromatic or olefinic methines and four nonprotonated carbons [including one ketone carbonyl at *δ*_C_ 197.4 (C-7)]. Among them, one benzene ring, an acetyl group and four olefinic carbons occupied seven degrees of unsaturation. Hence, the remaining one degree of unsaturation can only be due to the presence of one ring. The key HMBC correlations (Fig. 2) from H-2 to C-3/C-4/C-5 and from H-5 to C-2/C-3/C-4 and from H-8′ to C-2/C-5 demonstrated the existence of a pyrrole nucleus. The ^1^H-^1^H COSY correlations (Fig. 2) between H_2_-7′ and H_2_-8′ and the key HMBC correlations from H-7′ to C-1′/C-2′/C-6′, H-8′ to C-2/C-5/C-7′ showed the phenylethyl was attached to the nitrogen atom. In addition, the acetyl can be confirmed by the key HMBC correlation from H-8 to C-7. Finally, the locations of the two substituents (an acetyl group and an ethoxy group) on the pyrrole nucleus were also confirmed at C-3, C-4 based on the HMBC correlations from H-8 to C-3 and from H-6 to C-3/C-4/C-5. Compound **1** was, therefore, established as albifipyrrol A, as depicted.

**Table 1:** ^1^H and ^13^C NMR data (*δ* in ppm and *J* in Hz) of compounds **1**–**3**

No.	**1**		**2**		**3**	
	*δ*_C_^a^	*δ*_H_^b^, mult (*J*)	*δ*_C_^a^	*δ*_H_^b^, mult (*J*)	*δ*_C_^c^	*δ*_H_^d^, mult (*J*)
2	131.8,CH	7.40, d (2.2)	130.9,CH	7.36, d (2.2)	131.6,CH	7.61, d (2.2)
3	123.9,C		123.5,C		124.3,C	
4	126.7,C		123.1,C		127.0,C	
5	122.4,CH	6.72, d (2.2)	123.7,CH	6.70, d (2.2)	122.4,CH	6.77, d (2.2)
6	58.5,CH_2_	4.56, s	68.6,CH_2_	4.54, d (0.8)	58.5,CH_2_	4.59, d (0.8)
6-OMe			58.2,CH_3_	3.34, s		
7	197.4,C		196.4,C		197.5,C	
8	26.9,CH_3_	2.32, s	27.3,CH_3_	2.29, s	27.0,CH_3_	2.40, s
1'	139.5,C		139.5,C		49.9,CH_2_	3.99, t (7.0)
2'	129.9,CH	7.13, d (7.8)	129.9,CH	7.12, d (7.8)	27.4,CH_2_	2.08, m
3'	129.6,CH	7.24–7.28, m	129.6,CH	7.23–7.27, m	31.4,CH_2_	2.32, t (7.3)
4'	127.7,CH	7.18–7.22, m	127.7,CH	7.18–7.22, m	174.8,C	
5'	129.6,CH	7.24–7.28, m	129.6,CH	7.23–7.27, m	52.2,CH_3_	3.65, s
6'	129.9,CH	7.13, d (7.8)	129.9,CH	7.12, d (7.8)		
7'	38.6,CH_2_	3.06, t (7.1)	38.7,CH_2_	3.06, t (7.1)		
8'	52.5,CH_2_	4.16, t (7.1)	52.5,CH_2_	4.16, t (7.1)		

^a^Recorded at 150 MHz, Recorded in Methanol-*d*_4_

^b^Recorded at 600 MHz, Recorded in Methanol-*d*_4_

^c^Recorded at 126 MHz, Recorded in Methanol-*d*_4_

^d^Recorded at 500 MHz, Recorded in Methanol-*d*_4_

Compound **2** was obtained as yellow oil. The molecular ion peak of HR-ESI–MS was at *m/z* 280.1306 [M + Na]^+^ (calcd for 280.1313), which deduced that the molecular formula of compound **2** was C_16_H_19_NO_2_, with eight degrees of unsaturation. The ^1^H-NMR and ^13^C-NMR data (Table 1) suggested **2** was similar to **1** and the only observed difference was that the hydroxy group in **1** was replaced by a methoxy group in **2**. This change can be confirmed by the key HMBC correlations (Fig. 2) from H_3_-OMe to C-6. Compound **2** was, therefore, established as albifipyrrol B, as depicted.

Compound **3** was obtained as yellow oil. The molecular ion peak of HR-ESI–MS is at *m/z* 262.1046 [M + Na]^+^ (calcd for 262.1055), which indicated that the molecular formula of compound **3** is C_12_H_17_NO_4_, with five degrees of unsaturation. The ^1^H-NMR (Table 1) and HSQC spectrum of **3** revealed **3** has the same pyrrole ring as **1** and the major difference was the substituents on nitrogen. The ^1^H-NMR showed the signals of one methoxy [*δ*_H_ 3.65 (3H, s, H-5′); *δ*_C_ 52.2 (C-5′)], one carboxyl group [*δ*_C_ 174.8 (C-4′)], three methylenes [*δ*_H_ 2.32 (2H, t, *J* = 7.3 Hz, H-3′), 2.08 (2H, m, H-2′), 3.99 (2H, t, *J* = 7.0 Hz, H-1′); *δ*_C_ 31.4 (C-3′), 27.4 (C-2′), 49.9 (C-1′)]. The methyl butyrate unit was established by the ^1^H-^1^H COSY correlations between H_2_-1′, H_2_-2′ and H_2_-3′ and the key HMBC correlations (Fig. 2) from H-5′ to C-4′ and from H-3′ to C-4′. Finally, the attachment position of the methyl butyrate residue to the pyrrole ring was defined on the basis of HMBC correlations between H-1′and C-2/C-5. Compound **3** was, therefore, established as albifipyrrol C, as depicted.

All new compounds were evaluated for their in vitro inhibition activities on concanavalin A (Con A) induced T cell proliferation and lipopolysaccharide (LPS) induced B cell proliferation. Compound **2** exhibited certain inhibition specifically against the LPS-induced proliferation of B lymphocyte cells with IC_50_ value 16.6 μM (Table 2).

**Table 2:** Immunosuppressive tests of compounds **1–3**

Compound	ConA-induced T-cell proliferation	LPS-induced B-cell proliferation
	IC_50_(*μ*M)	IC_50_(*μ*M)
**1**	NA^a^	NA^a^
**2**	NA^a^	16.16
**3**	NA^a^	NA^a^
**CsA**^b^	0.05	0.37

^a^NA: no activity

^b^Positive control

## Experimental section

### General experimental procedures

1D and 2D NMR spectra were recorded on Bruker DXR-600 instrument (600 and 150 MHz) and Bruker DXR-500 instrument (500 and 126 MHz). The UV data were detected by Hitachi UH5300 spectrophotometer (Hitachi, Kyoto, Japan). IR spectra were conducted on IRT racer-100 (SHIMADZU, Kyoto, Japan) with KBr pellets. HR-ESI–MS data were obtained on a UPLC-Q Exactive MS system (Thermo Fisher, Santa Clara, CA, USA). The packing for column chromatography (CC) is silica gel (200–300 mesh, Qingdao Haiyang Chemical Co., Ltd., Qingdao, China) or Sephadex LH-20 (Amersham Biosciences, Upssala, Sweden). The semi-prepared HPLC was carried out on an Agilent Technologies 1260 Infinity II system with a diode array detector. And the chromatographic column was C_18_ reversed phase column (5 μm, 10 × 250 mm) (Agela, Tianjin, China).

### Fungal material

The strain were isolated from the roots of *C. chinensis* collected from Enshi, Hubei province, and was identified as *Albifimbria viridis* via 18S rDNA sequences and deposited at South-Central University for Nationalities, China. The sequence data for this strain had been submitted to the DDBJ/EMBL/Genbank with accession No. MT110686.1.

### Extraction and isolation

The fungus *Albifimbria viridis* was fermented on solid rice medium (100 g of rice, 100 mL of water, in each 500 mL culture flask) and was cultured at 30 °C for one month. The fermented material was soaked in absolute methanol (20 L × 4). The combined extracts were evaporated under reduced pressure to afford an crude extract, which was further dissolved in water and extracted three times with EtOAc (10 L × 4) to yield 110 g of the extract. The crude extract was subjected to silica gel column chromatography (petroleum ether: ethyl acetate, 15:1 to 0:1; ethyl acetate: methyl alcohol, 15:1 to 0:1) to yield six fractions (A‒F). Fraction B (6 g) was separated into eight sub-fractions (B_1_‒B_8_) by ODS MPLC. The eluent is composed of methyl alcohol: H_2_O (from 10:90 to 100:0, v/v). Fraction B_3_ was purified by semi-preparative HPLC (CH_3_CN/H_2_O = 55:45, v/v) to give compound **1** (4.7 mg, *t*_R_ = 18.7 min). Fraction C (5 g) was isolated from Sephadex LH-20 eluting with MeOH and purified by semi-preparative HPLC (CH_3_CN/H_2_O = 40:60, v/v) to obtain compound **2** (1.3 mg, *t*_R_ = 25 min). Fraction D (7.5 g) was isolated by Sephadex LH-20 column chromatography (MeOH) to obtain six sub-fractions (D_1_‒D_6_). Fraction D_5_ was purified by semi-preparative HPLC (CH_3_CN/H_2_O from 25:75 to 45:55 in 20 min, v/v) to yield compound **3** (2.4 mg, *t*_R_ = 13.2 min).

### Spectroscopic data of compounds

#### Albifipyrrol A (1)

Yellow oil. UV (MeOH) λ_max_ (log ε): 210 (1.97). HR-ESI–MS *m/z* found 266.1150 [M + Na]^+^ (Calcd for C_15_H_17_NO_2_Na, 266.1157). IR (KBr) ν_max_ (cm^–1^): 3401, 2949, 2837, 1655, 1450, 1117, 1024. ^1^H and ^13^C-NMR see (Table 1).

#### Albifipyrrol B (2)

Yellow oil. UV (MeOH) λmax (log ε): 210 (1.82). HR-ESI–MS *m/z* found 280.1306 [M + Na]^+^ (Calcd for C_16_H_19_NO_2_Na, 280.1313). IR (KBr) ν_max_ (cm^–1^): 3364, 2945, 2833, 1670, 1452, 1119, 1032. ^1^H and ^13^C-NMR see (Table 1).

#### Albifipyrrol C (3)

Yellow oil. UV (MeOH) λ_max_ (log ε): 255 (2.01). HR-ESI–MS *m/z* found 262.1046 [M + Na]^+^ (Calcd for C_12_H_17_NO_4_Na, 262.1055). IR (KBr) ν_max_ (cm^–1^): 3400, 2950, 1734, 1632, 1526, 1157. ^1^H and ^13^C-NMR see (Table 1).

### Immunosuppressive activities assay

Fresh spleen cells were obtained from female BALB/c mice (6–8 weeks old). Spleen cells (1 × 10^6^ cells) were cultured in triplicate on a 96-well plate for 48 h at 37 °C in a humidified incubator containing 5% CO_2_ (with or without different concentrations of compounds). During the last 8 h of culture, a certain amount of CCK-8 was added to each well. At the end of culture, the OD values at 450 nm was measured by a bio-RAD 650 microplate reader. Cells with viability above 85% were further screened for their inhibitory activity against T and B lymphocytes. The 5 × 10^5^ spleen cells were cultured at the same conditions as those mentioned above. T cell or B cell proliferation was induced with 10 µg ml^−1^ of LPS or 5 µg ml^−1^ of ConA, respectively. Proliferation was assessed in terms of uptake of [^3^H]-thymidine during 8 h of pulsing with 25 µL/well of [^3^H]-thymidine, and then cells will be harvested onto glass fiber filters. The incorporated radioactivity was counted using a Beta scintillation counter (MicroBeta Trilux, PerkinElmer Life Sciences). Cells treated without any stimuli were used as negative control. The immunosuppressive activity of each compound was expressed as the concentration of compound that inhibited ConA induced T cell proliferation or LPS-induced B cell proliferation to 50% (IC_50_) of the control value. Both the cytotoxicity and proliferation assessment repeated twice. Cyclosporin A (CsA) an immunosuppressive agent, was used as a positive control (Table 2; Additional file 1: Figs. S1–S24).

## Supplementary Information

**Additional file 1: Figure S1.** ^1^H NMR (600 MHz, CD3OD) spectrum of compound 1. **Figure S2.** ^13^C NMR (150 MHz, CD3OD) spectrum of compound 1. **Figure S3.** HSQC spectrum of compound 1. **Figure S4.** COSY spectrum of compound 1. **Figure S5.** HMBC spectrum of compound 1. **Figure S6.** HRESIMS of compound 1**. Figure S7.** UV spectrum of compound 1. **Figure S8.** IR spectrum of compound 1. **Figure S9.** ^1^H NMR (600 MHz, CD3OD) spectrum of compound 2. **Figure S10.** ^13^C NMR (150 MHz, CD3OD) spectrum of compound 2. **Figure S11.** HSQC spectrum of compound 2. **Figure S12.** COSY spectrum of compound 2. **Figure S13.** HMBC spectrum of compound 2. **Figure S14.** HRESIMS of compound 2. **Figure S15.** UV spectrum of compound 2. **Figure S16.** IR spectrum of compound 2. **Figure S17.** ^1^H NMR (500 MHz, CD3OD) spectrum of compound 3. **Figure S18.** ^13^C NMR (126 MHz, CD3OD) spectrum of compound 3. **Figure S19.** HSQC spectrum of compound 3. **Figure S20.** COSY spectrum of compound 3. **Figure S21.** HMBC spectrum of compound 3. **Figure S22.** HRESIMS of compound 3. **Figure S23.** UV spectrum of compound 3. **Figure S24.** IR spectrum of compound 3.
