# Supplementary material for: Three new pyrrole alkaloids from the endophytic fungus Albifimbria viridis
Source: Nat Prod Bioprospect. 2022 Feb 24;12(1):5. doi: 10.1007/s13659-022-00327-2 (PMC8866607; doi:10.1007/s13659-022-00327-2)
Supplement: Supplementary file 1 — Additional file 1: Figure S1. 1H NMR (600 MHz, CD3OD) spectrum of compound 1. Figure S2. 13C NMR (150 MHz, CD3OD) spectrum of compound 1. Figure S3. HSQC spectrum of compound 1. Figure S4. COSY spectrum of compound 1. Figure S5. HMBC spectrum of compound 1. Figure S6. HRESIMS of compound 1. Figure S7. UV spectrum of compound 1. Figure S8. IR spectrum of compound 1. Figure S9. 1H NMR (600 MHz, CD3OD) spectrum of compound 2. Figure S10. 13C NMR (150 MHz, CD3OD) spectrum of compound 2. Figure S11. HSQC spectrum of compound 2. Figure S12. COSY spectrum of compound 2. Figure S13. HMBC spectrum of compound 2. Figure S14. HRESIMS of compound 2. Figure S15. UV spectrum of compound 2. Figure S16. IR spectrum of compound 2. Figure S17. 1H NMR (500 MHz, CD3OD) spectrum of compound 3. Figure S18. 13C NMR (126 MHz, CD3OD) spectrum of compound 3. Figure S19. HSQC spectrum of compound 3. Figure S20. COSY spectrum of compound 3. Figure S21. HMBC spectrum of compound 3. Figure S22. HRESIMS of compound 3. Figure S23. UV spectrum of compound 3. Figure S24. IR spectrum of compound 3. [file 13659_2022_327_MOESM1_ESM.docx]

**Additional Information for**

**Three new pyrrole alkaloids from the endophytic fungus *Albifimbria viridis***

Pan-Pan Wei, Jia-Cheng Ji, Xu-Jun Ma, Zheng-Hui Li, Hong-Lian Ai*, Xin-Xiang Lei*, Ji-Kai Liu*

School of Pharmaceutical Sciences, South-Central University for Nationalities, Wuhan 430074, People’s Republic of China

*Corresponding author: aihonglian@mail.scuec.edu.cn; [xxlei@mail.scuec.edu.cn](mailto:xxlei@mail.scuec.edu.cn); liujikai@mail.scuec.edu.cn

**List of additional information**

**Figure S1. ^1^H NMR (600 MHz, CD3OD) spectrum of compound 1**

**Figure S2. ^13^C NMR (150 MHz, CD3OD) spectrum of compound 1**

**Figure S3. HSQC spectrum of compound 1**

**Figure S4. COSY spectrum of compound 1**

**Figure S5. HMBC spectrum of compound 1**

**Figure S6. HRESIMS of compound 1**

**Figure S7. UV spectrum of compound 1**

**Figure S8. IR spectrum of compound 1**

**Figure S9. ^1^H NMR (600 MHz, CD3OD) spectrum of compound 2**

**Figure S10. ^13^C NMR (150 MHz, CD3OD) spectrum of compound 2**

**Figure S11. HSQC spectrum of compound 2**

**Figure S12. COSY spectrum of compound 2**

**Figure S13. HMBC spectrum of compound 2**

**Figure S14. HRESIMS of compound 2**

**Figure S15. UV spectrum of compound 2**

**Figure S16. IR spectrum of compound 2**

**Figure S17. ^1^H NMR (500 MHz, CD3OD) spectrum of compound 3**

**Figure S18. ^13^C NMR (126 MHz, CD3OD) spectrum of compound 3**

**Figure S19. HSQC spectrum of compound 3**

**Figure S20. COSY spectrum of compound 3**

**Figure S21. HMBC spectrum of compound 3**

**Figure S22. HRESIMS of compound 3**

**Figure S23. UV spectrum of compound 3**

**Figure S24. IR spectrum of compound 3**

**Figure S1. ^1^H NMR (600 MHz, CD3OD) spectrum of compound 1**

**Figure S2. ^13^C NMR (150 MHz, CD3OD) spectrum of compound 1**

**Figure S3. HSQC spectrum of compound 1**

**Figure S4. COSY spectrum of compound 1**

**Figure S5. HMBC spectrum of compound 1**

**Figure S6. HRESIMS of compound 1**

**
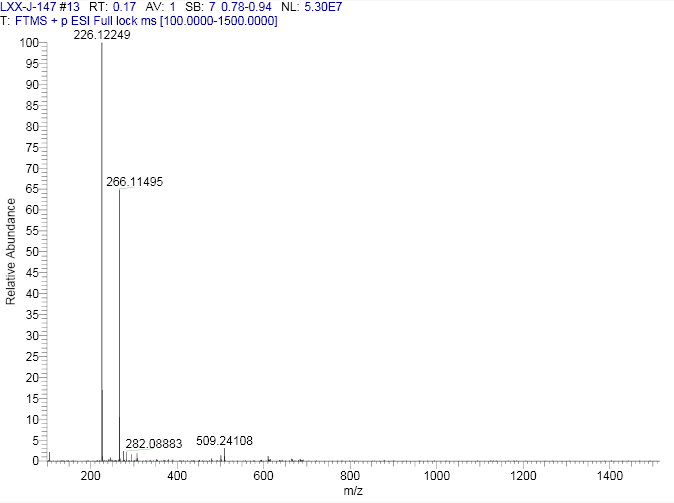
**

**Figure S7. UV spectrum of compound 1**

**
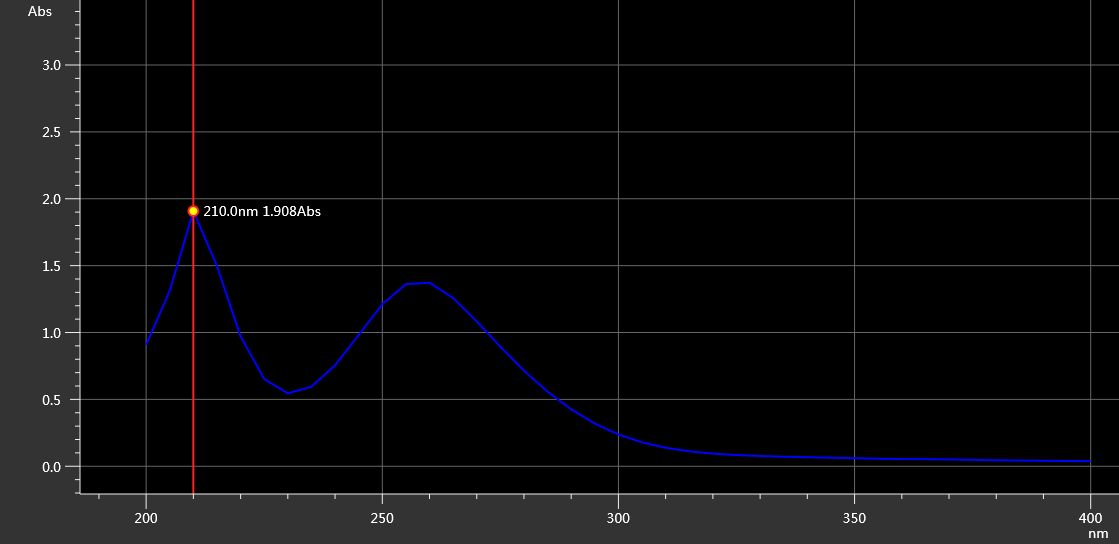
**

**Figure S8. IR spectrum of compound 1**

**
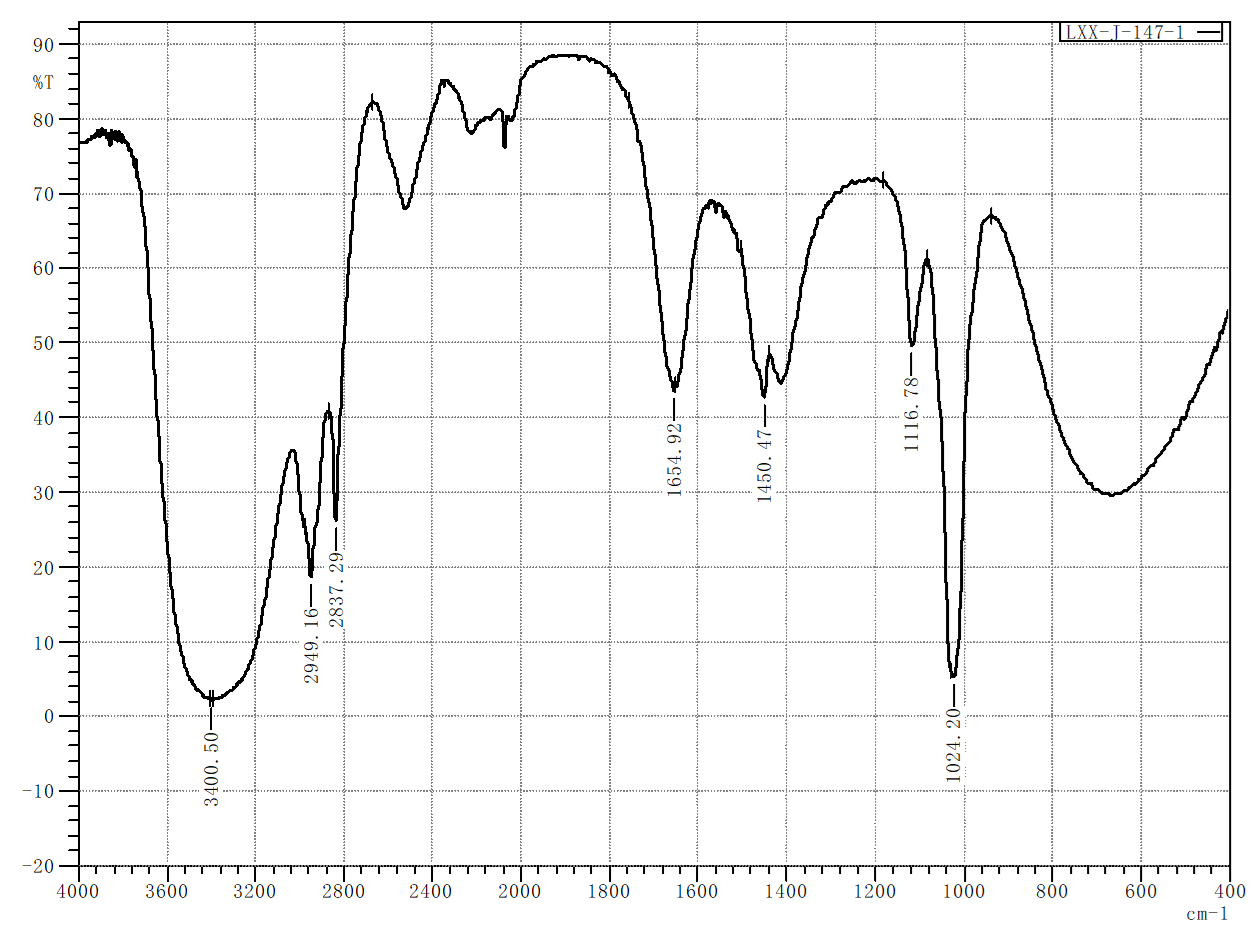
**

**Figure S9. ^1^H NMR (600 MHz, CD3OD) spectrum of compound 2**

**Figure S10. ^13^C NMR (150 MHz, CD3OD) spectrum of compound 2**

**Figure S11. HSQC spectrum of compound 2**

**Figure S12. COSY spectrum of compound 2**

**Figure S13. HMBC spectrum of compound 2**

**Figure S14. HRESIMS of compound 2**

**
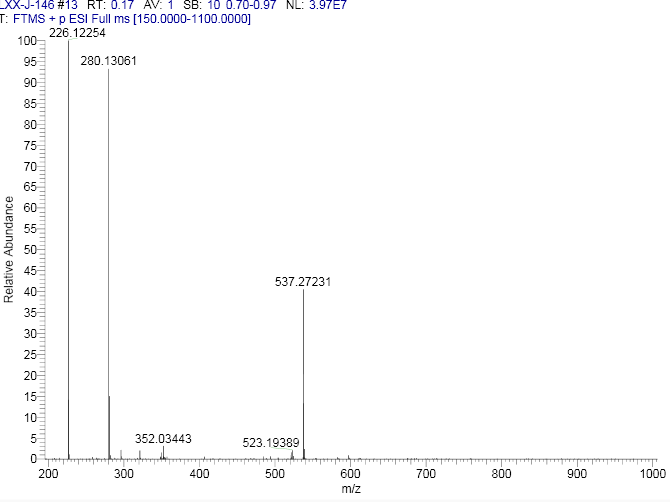
**

**Figure S15. UV spectrum of compound 2**

**
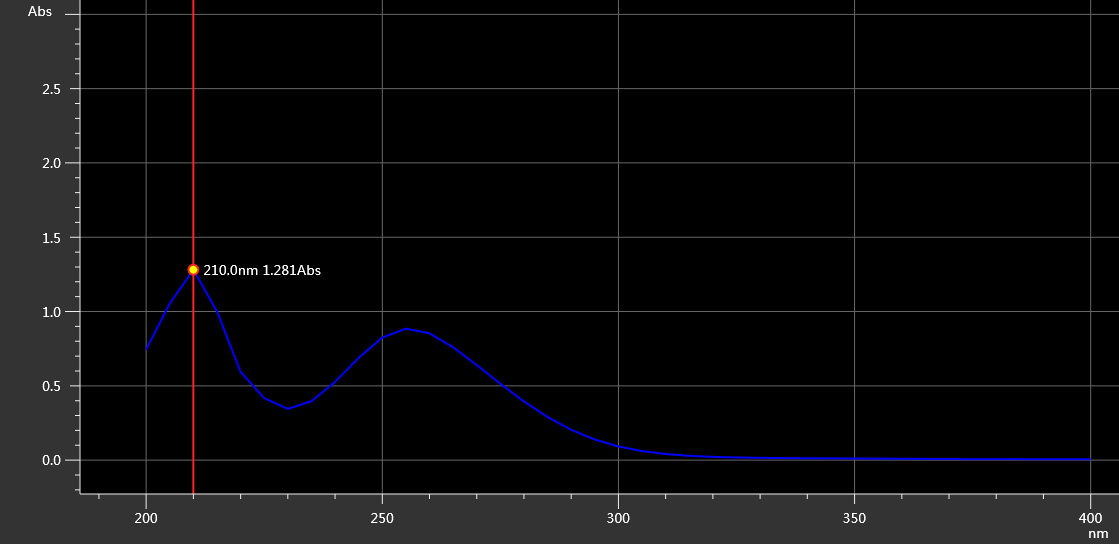
**

**Figure S16. IR spectrum of compound 2**

**
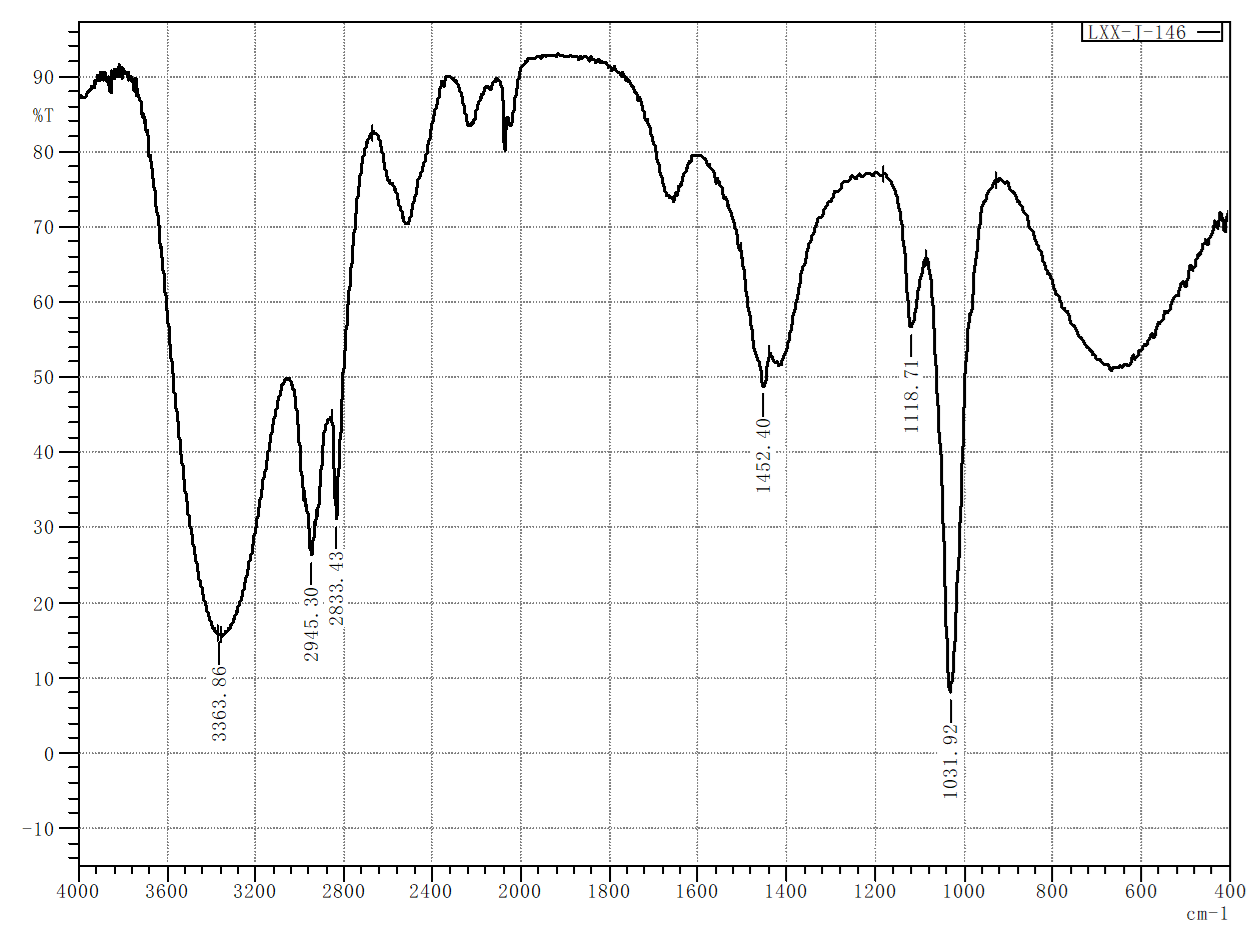
**

**Figure S17. ^1^H NMR (500 MHz, CD3OD) spectrum of compound 3**

**Figure S18. ^13^C NMR (126 MHz, CD3OD) spectrum of compound 3**

**Figure S19. HSQC spectrum of compound 3**

**Figure S20. COSY spectrum of compound 3**

**Figure S21. HMBC spectrum of compound 3**

**Figure S22. HRESIMS of compound 3**

**
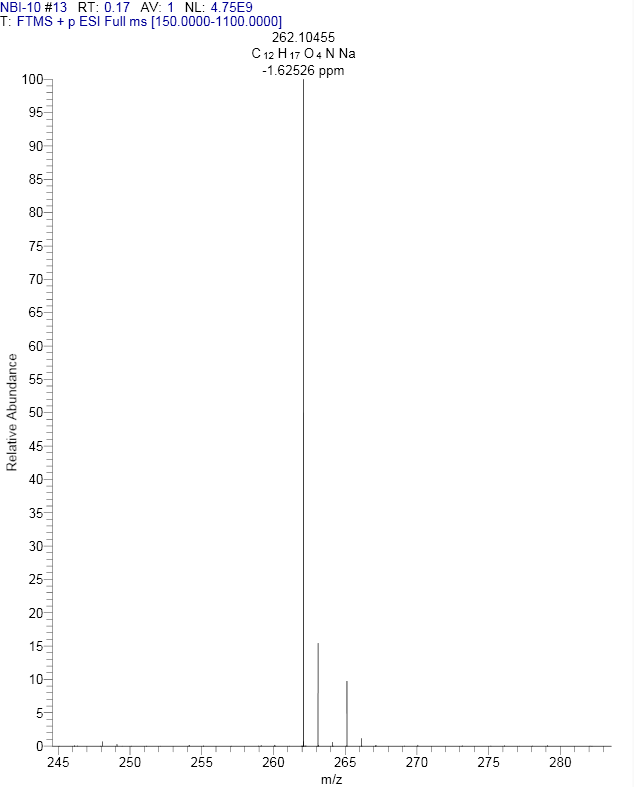
**

**Figure S23. UV spectrum of compound 3**

**
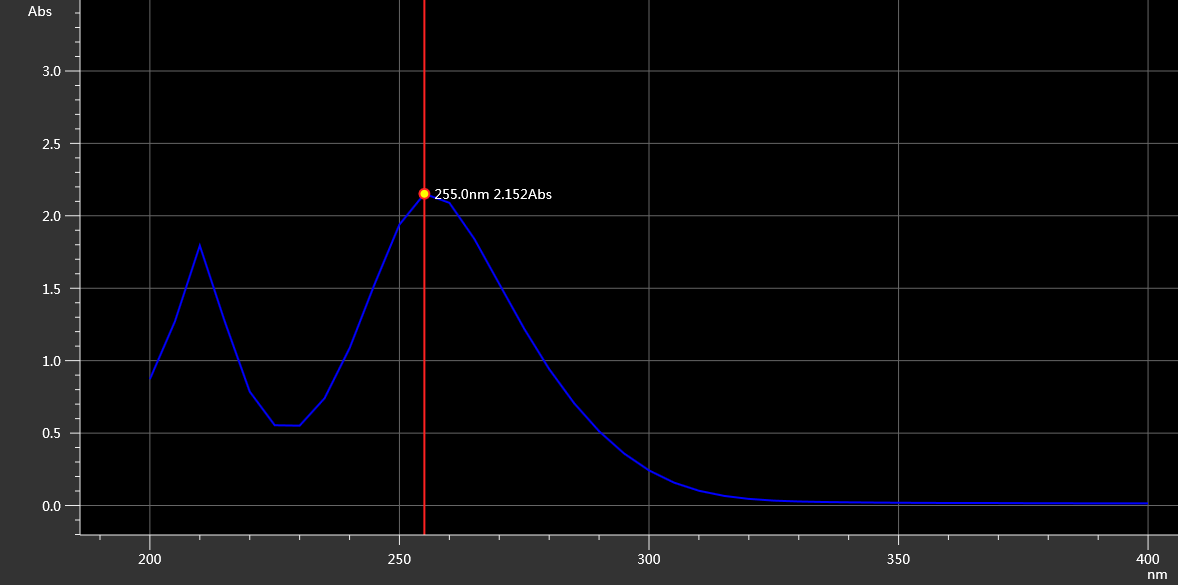
**

**Figure S24. IR spectrum of compound 3**

**
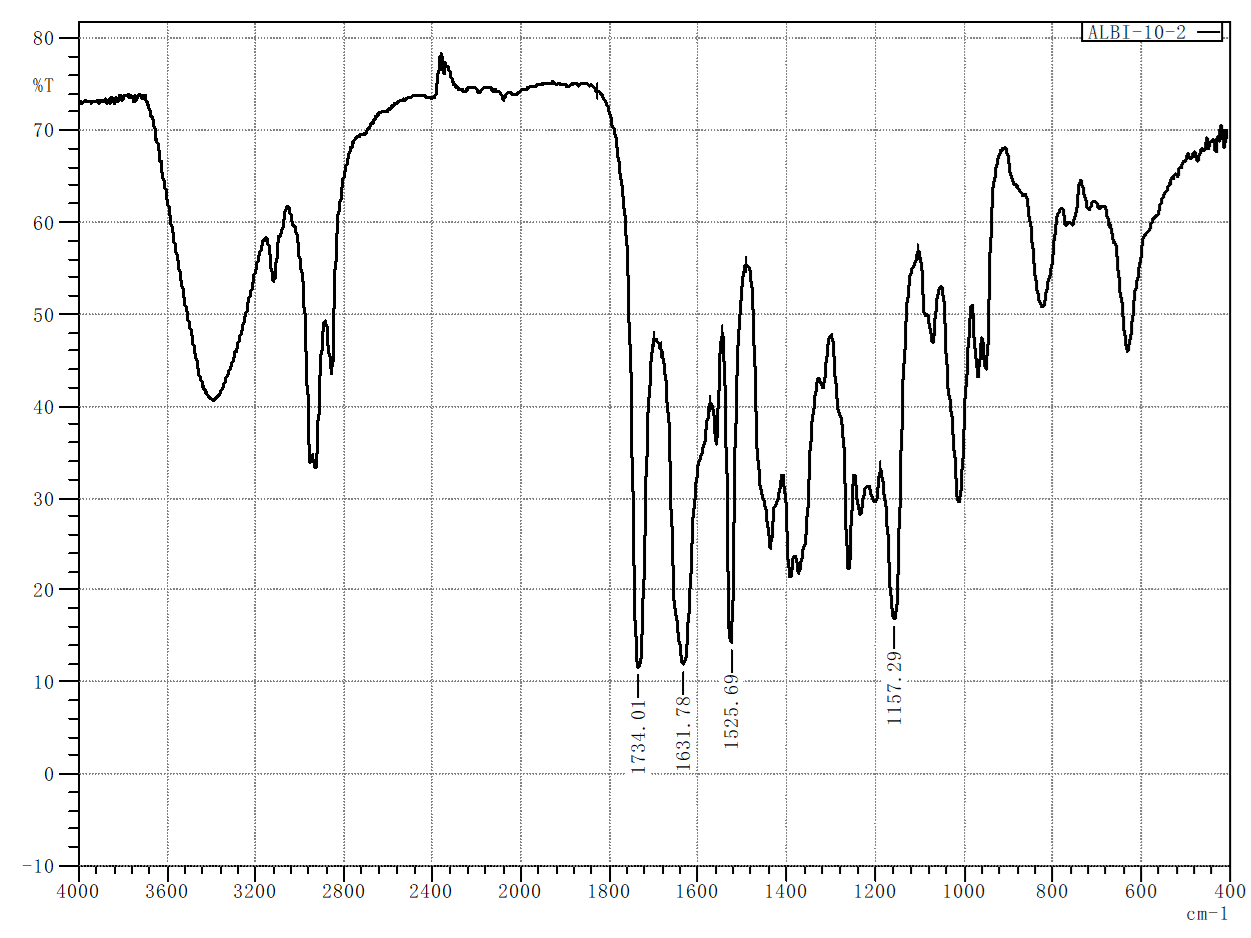
**
